# A low direct electrical signal attenuates oxidative stress and inflammation in septic rats

**DOI:** 10.1371/journal.pone.0257177

**Published:** 2021-09-09

**Authors:** Savas Ustunova, Ebru Haciosmanoglu, Huri Bulut, Birsen Elibol, Aysu Kilic, Rumeysa Hekimoglu, Serkan Tunc, Rabia Atmaca, Irem Kaygusuz, Sevil Tunc, Gulcin Beyza Tunc, Ismail Meral

**Affiliations:** 1 Department of Physiology, School of Medicine, Bezmialem Vakif University, Istanbul, Turkey; 2 Department of Biophysics, School of Medicine, Bezmialem Vakif University, Istanbul, Turkey; 3 Department of Biochemistry, School of Medicine, Istinye University, Istanbul, Turkey; 4 Department of Medical Biology, School of Medicine, Bezmialem Vakif University, Istanbul, Turkey; 5 Department of Histology & Embryology, School of Medicine, Bezmialem Vakif University, Istanbul, Turkey; 6 AVB Biotech, Istanbul, Turkey; University of Messina, ITALY

## Abstract

Electrical stimulation is proposed to exert an antimicrobial effect according to studies performed using bacterial and cell cultures. Therefore, we investigated the effects of electrification on inflammation in septic rats. Twenty-eight male Wistar albino rats were divided into 4 groups: healthy control (C), electrified healthy (E), sepsis (S), and electrified sepsis (SE) groups. *Staphylococcus aureus* (1 x 10^9^ colonies) in 1 ml of medium was intraperitoneally injected into rats to produce a sepsis model. The rats in the E and SE groups were exposed to a low direct electrical signal (300 Hz and 2.5 volts) for 40 min and 1 and 6 h after bacterial infection. Immediately after the second electrical signal application, blood and tissue samples of the heart, lung, and liver were collected. An antibacterial effect of a low direct electrical signal was observed in the blood of rats. The effects of electrical signals on ameliorating changes in the histological structure of tissues, blood pH, gases, viscosity and cell count, activities of some important enzymes, oxidative stress parameters, inflammation and tissue apoptosis were observed in the SE group compared to the S group. Low direct electrical signal application exerts antibacterial, antioxidant, anti-inflammatory and antiapoptotic effects on septic rats due to the induction of electrolysis in body fluids without producing any tissue damage.

## Introduction

Inflammation is a complex biological process that protects against harmful stimuli and affects many organs, and it has a role in disease progression unless it is properly controlled. The acute inflammatory response is critical in host defense, but, if not treated, can lead to chronic inflammation associated with many human diseases. Sepsis is one of these conditions because it is a form of widespread inflammation in the body. Sepsis is defined as an unusual pattern of response by the immune system to injury or bacterial infection.

During the early phase of sepsis, receptors such as Toll-like receptors on the surface of macrophages recognize molecules related to the pathogen; then, proinflammatory cytokines, such as tumor necrosis factor (TNF), interleukin-1β (IL-1β) and IL6, are secreted from macrophages to induce systemic inflammation. In addition to these proinflammatory cytokines, upregulation of the levels of C-reactive protein (CRP) and procalcitonin (PCT) are other biomarkers of sepsis. During the progression of sepsis, widespread organ dysfunction (lung, liver, kidney, and brain) is initiated, which is called severe sepsis. Finally, septic shock occurs due to cardiovascular disruptions [1]. In addition, sepsis-dependent organ dysfunction exacerbates reactive oxygen species (ROS) production because oxidative stress is one of the factors that stimulates proinflammatory cytokine secretion [2]. Therefore, new therapeutic approaches are urgently needed for diseases in which the inflammatory response contributes to progressive loss of organ function [3].

In recent years, drug-free treatment for diseases has begun to be preferred because it has fewer side effects. For instance, physical interventions, such as electrical or magnetic stimulation, with varying parameters produce positive results for acute or chronic nerve injuries. The biological basis of electrical or magnetic stimulation is mainly based on alterations in protein synthesis, ion channel regulation, and growth factor secretion [4]. Due to these properties, an electric current is also used in medicine for pain relief (TENS), a reduction in inflammation, and wound healing [5].

Electrification is a technique that has the potential to destroy pathogens, viruses, bacteria, parasites, germs and fungi that disrupt our health by impairing our immune system [6, 7]. Previously, the antimicrobial effects of electrification on water, milk, and bacterial and cell cultures were documented *in vitro* [8, 9]. In addition, most acquired immunodeficiency syndrome (AIDS) viruses lose their infectious abilities after exposure to a very low electrical current *in vitro* [10]. When the blood is repeatedly exposed to electric current, AIDS and hepatitis viruses are almost undetectable, without damaging the blood cells. Subsequently, the same effect was achieved in humans by placing the electrodes on the blood vessel paths and exposing them to a very low electric current [11]. Although such a protocol was developed, interestingly, no *in vivo* study has been conducted on electrical stimulation to date, except for wound healing. In these wound healing studies, bacterial inhibition was detected in infected wounds in humans following electrical stimulation [12–14]. Although electrical stimulation directly or indirectly produces its antibacterial effect on infected wounds, its exact mechanism is still not fully understood. The direct effect may occur because the electric current disrupts the integrity of the bacterial membrane or induces the electrolysis of molecules on the bacterial cell surface, which is the most likely cause of the antibacterial effects of electrical stimulation [15, 16]. Temperature and pH changes may also be indirect effects of electrical stimulation [17, 18]. However, these changes may not be the primary cause of the antibacterial effects of electrical stimulation [14]. Therefore, the current study was designed to investigate the *in vivo* effects of a low direct electrical signal on the inflammatory response, blood bacterial count, pH, gases, viscosity, cell count, some biochemical parameters, oxidative stress and tissue damage in a rat bacterial sepsis model.

## Materials and methods

### Preparation of the bacterial suspension

A commercially available *Staphylococcus aureus* standard strain (ATCC-10390, USA) was used in the study. Before the bacterial injection, passages were taken from the lyophilized strains stored at -20°C, and an incubation was carried out at appropriate times. Identification was performed at certain time intervals using the VITEK 2 Compact 30 automatic micro identification system and VITEK MS mass spectrophotometer (bioMérieux, Lyon, France) to ensure that no contamination or structural change occurred in standard strains. As a result of the identification, the strains showed similarity at a rate of 99.9%. In addition, only bacteria obtained from the first passages were always used to prevent the standard strain from mutating and losing its pathogenicity. Standard *S*. *aureus* strains were in the logarithmic phase of growth during the experiment. Therefore, passages were taken 18–24 hours before injection and incubated at 37°C during this period. After incubation, the appropriate density of the bacterial suspension (1x10^9^ CFU/ml) was prepared using the DensiCHEK plus (bioMérieux, Lyon, France) device according to the McFarland turbidity system. Sterile ACILA®LAL Reagent Water (LRW, Opelstrassee 14, Mörfelden-Walldorf, Germany) was used to prepare the suspensions. The prepared suspensions were drawn into 1 ml injectors under sterile conditions and were ready for administration. At the end of the experiment, microbiological examinations were performed using the culture method in blood samples to determine the reliability of our sepsis model.

### Determination of culturable bacteria

Blood samples (100 μl) collected 1 and 6 hours after bacterial injection were pre-enriched for 3 hours in 10 ml commercial TSB tubes (BD, BBL Trypticase Soy Broth, Franklin Lakes, New Jersey, USA). At the end of 3 hours, 100 μl of the solution was pipetted onto the center of the surface of three different Mannitol Salt Agar plates (Merck-Millipore, Darmstadt, Germany) using the spread plate method. The plates were incubated at 37°C for 72 hours. At the end of this period, colony forming units in plates were counted, and the mean results were used for statistical analysis.

### Device used to apply the electric current

This newly developed low electrical signal application device (Dr. Biolyse) is an electron accelerator that can be used to decompose chemicals in body fluids (Turkish Patent Institute patent application numbers: 2021/006002, 2020/08818, 2020/14753, and 2020/14781). The principle of the device is that molecules decompose with the energy provided by the electrical signal, and new molecules are formed from the electrons supplied to the environment. In body fluids, 3 types of compounds have been identified whose molecular bonds are close to each other and thus could be easily decomposed and merged due to excitation: water (H_2_O), sodium chloride (NaCl) and potassium chloride (KCl). Therefore, before this experiment, we postulated that the necessary decomposition of these compounds and the formation of new compounds from the chemicals mentioned above in body fluids might be achieved by energy and electron transfer induced by low direct electrical signal exposure (electrolysis) without increasing the body temperature. We tested this hypothesis by applying a low direct electrical signal to the isotonic saline solution *in vitro* and thus increased the orbital velocity of electrons commonly used in covalent bonds to facilitate separation by resonating the average spin speed of the electron according to the speed at which it will break from the orbit. Using this method, highly efficient separation was achieved by applying a low direct electrical signal at a frequency of 300 Hz for the electron to reach a speed of 300.000 km/sec, which was the approximate speed of electrons after dissociation. Thus, H_2_O dissociated into hydrogen (H) and hydroxyl (OH) ions, NaCl dissociated into sodium (Na) and chloride (Cl) ions. As we continued the reaction, sodium hydroxide (NaOH), H and Cl were formed in the medium. After the formation of NaOH reached a certain level, instant hypochlorous acid (HOCl, a weak acid) formation occurred. Based on these results, the system rapidly balanced within certain periods. The equilibration reaction was NaOH + HOCl → NaCl + H_2_O + O.

**Fig 1:**
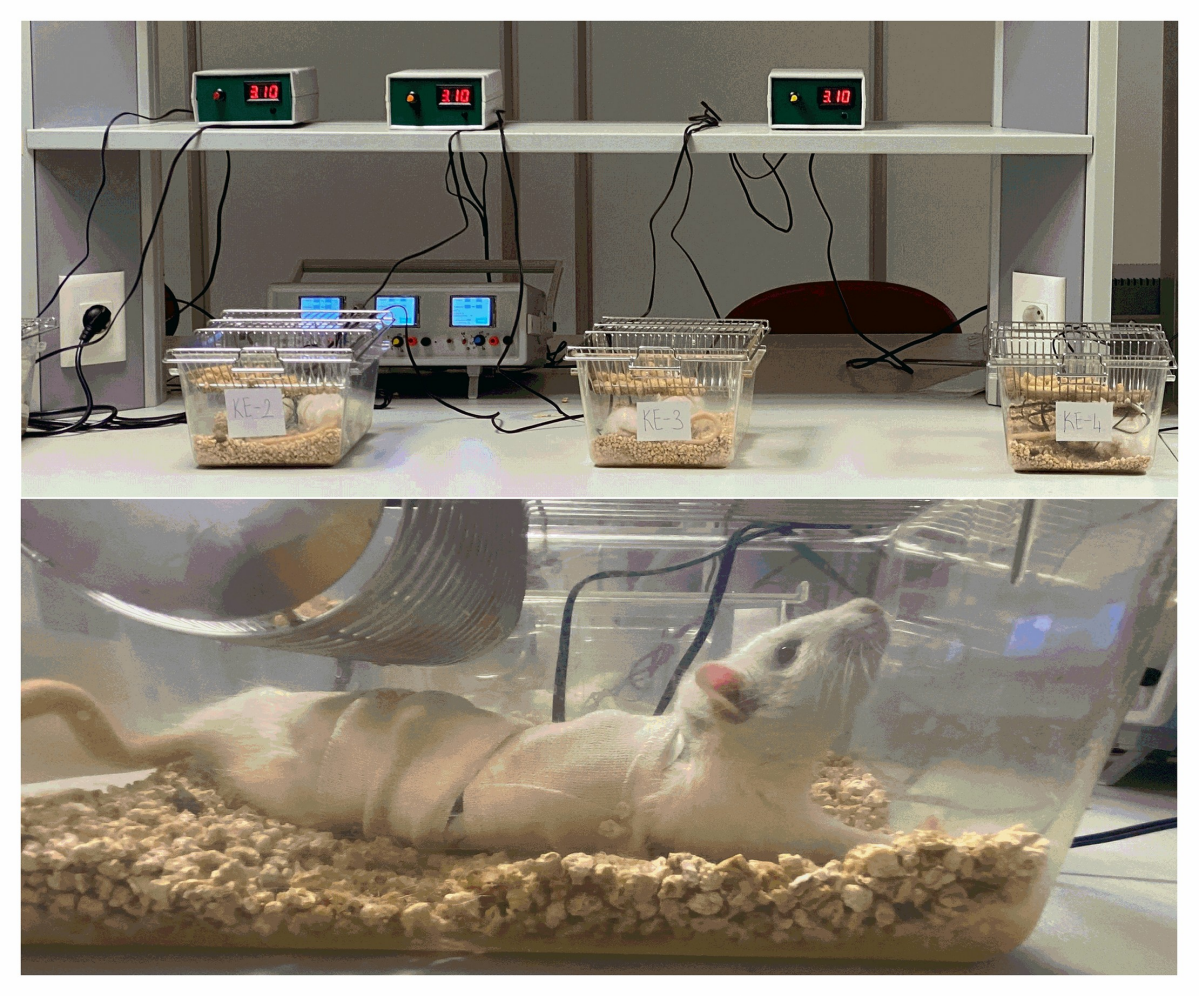
The Dr. Biolyse. Rats were exposed to a low electrical signal using the newly developed Dr. Biolyse device.

In this study, two pairs of carbon electrodes were placed on the lung and back and the abdomen and back to measure the resistance produced by the rat bodies exposed to the electrical signal. When the body resistance of rats was measured by applying a standard direct electrical signal, it ranged from 135.000–150.000 Ohms. Continuous application of this direct electrical signal would cause tissue damage over time due to the load it would create on the cells. However, when measured with the Dr. Biolyse device, which has a square-wave frequency featuring a direct electrical signal, the body resistance at a frequency of 300 Hz was in the range of 2.700–3.300 Ohms. This frequency of 300 Hz was selected according to our preliminary *in vitro* experiment, as the molecular separation occurred at the fastest level by providing the most efficient resonance on H_2_O, NaCl and KCl. With this frequency adjustment, the contact of the electrical signal with tissues was minimized, and the current applied to the body fluids was increased. Therefore, the electrical signal reached the target fluid environment over the body tissues with little resistance. The device limits itself by software (Fig 1).

### Animals and induction of sepsis

Based on the result of the power analysis (an average difference of 10 units and a standard deviation of 5 units at 80% power and 95% confidence level), a minimum of 7 rats was necessary for each group. Therefore, 28 male Wistar albino rats, weighing between 250–300 g, were used in this study. The rats were obtained from the Bezmialem Vakif University Experimental Animal Centre and housed under standard temperature (25 ± 1°C), humidity (50–60%), and dark-light conditions (12 h light/12 h dark cycle) and fed *ad libitum*.

This study was carried out in strict accordance with the recommendations in the Guide for the Care and Use of Laboratory Animals of the National Institutes of Health. The protocol and approved by the Bezmialem Vakif University Experimental Animals Ethical Committee (Date: 28.09.2020, No: 2020/145). All surgeries were performed under ketamine and xylazine anesthesia, and all efforts were made to minimize suffering.

**Fig 2:**
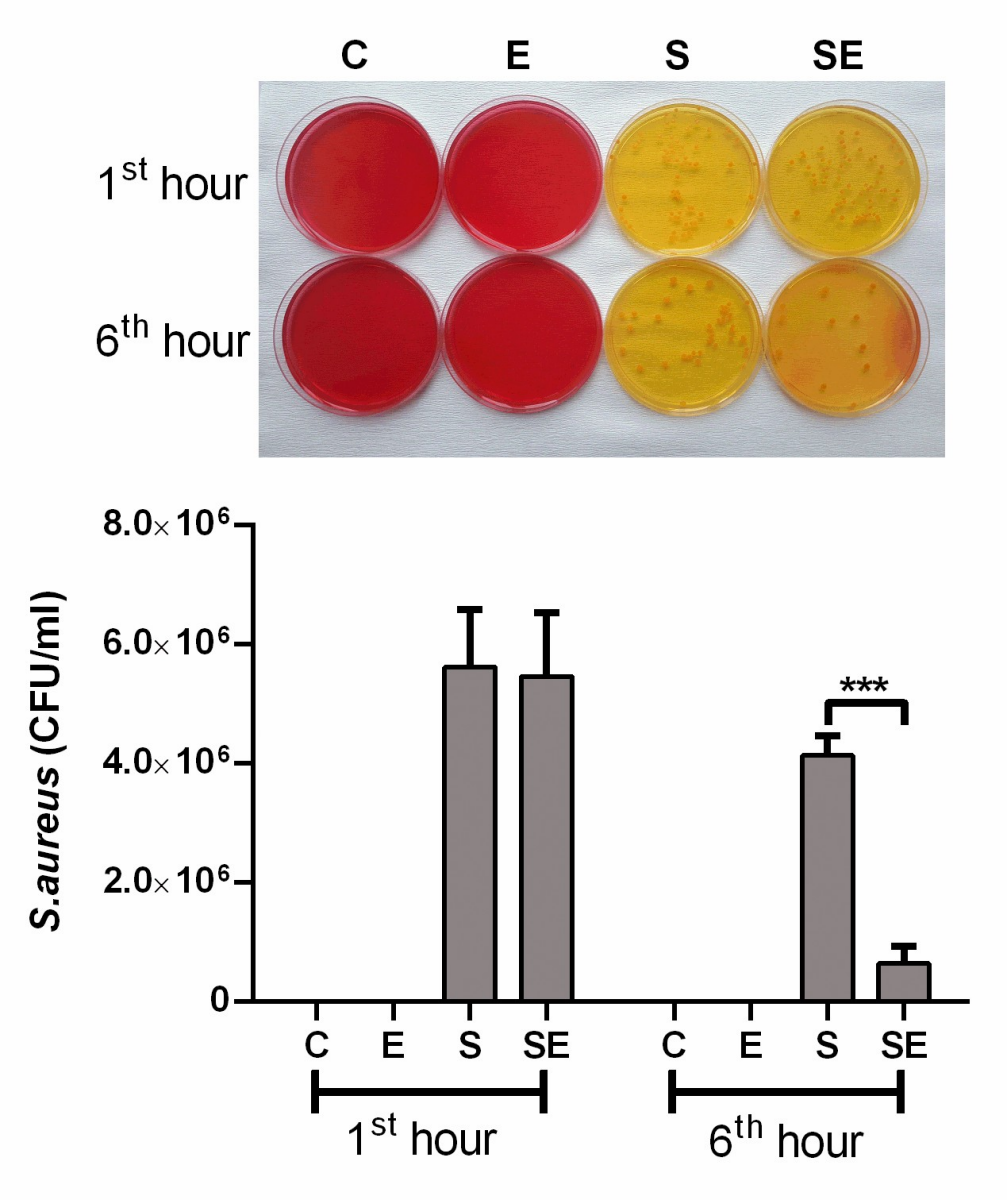
The number of bacteria measured during experiments. Bacterial counts of the healthy control (C) electrified healthy (E), sepsis (S) and electrified sepsis (SE) groups (n = 7). ***; *p*<0.001 statistically significant difference compared to the S group.

The rats were divided into 4 groups: healthy control (C), electrified healthy (E), sepsis (S), and electrified sepsis (SE) groups. Before the experiment, the abdominal and back areas of the rats were shaved with an electric razor for electrode placement. *Staphylococcus aureus* (1 x 10^9^ CFU) in 1 ml of LRW was administered (i.p.) to the animals to induce sepsis in the S and SE groups. The C and E groups were injected with 1 ml of LRW instead of *Staphylococcus aureus* medium [19]. Sepsis formation was confirmed by bacterial cultivation of 100 μl of blood taken from the jugular vein of the rats anesthetized with isoflurane 1 h after the bacteria or LRW injections (Fig 2).

Positive electrodes were placed on the chest and abdomen, and negative electrodes were placed on the back sides of chest and abdomen of rats to apply the low direct electrical signal (300 Hz and 2.5 volts) such that the electrical signal passed through the body for the electrolysis of the body fluids. The electrodes were fixed on the back and abdomen of the rats with a plaster that was completely wrapped around the body (Fig 1). The electrical signal was applied to rats in the E and SE groups for 40 min and 1 and 6 h after the bacterial injection. The electrodes were placed on the same areas of the rats in the C and S groups for 40 min without applying any electrical signal. At the end of this period, the rats were anesthetized with ketamine–xylazine (K, 100 mg/kg; X, 10 mg/kg, intraperitoneally), and cardiac blood samples were collected when animals were in deep sleep. Then, while rats were still under anesthesia, they were decapitated with a guillotine, and tissue samples were removed for further studies.

### Histopathological examination

The obtained tissues were fixed with 10% buffered formaldehyde for light microscopy. Samples stored in buffered formaldehyde were processed in a tissue processing machine under a fixed vacuum (Leica TP 1020, Germany). Tissues were embedded in paraffin wax. Three- to five-micrometer-thick paraffin sections were cut and then stained with hematoxylin–eosin for the histopathological examination. Each section was evaluated independently by two histologists who were blinded to the groups using the X10, X20 and X40 objectives. The images were captured using a Nikon digital camera (Eclipse 920248, USA).

### Whole blood measurements

The whole blood cell count was measured using a hematology analyzer (Abacus junior vet, Budapest, Hungary). The blood gases and pH were measured using a blood gas analyzer (ABL90 FLEX, Bronshoj, Denmark). Whole blood viscosity and shear stress were measured at a shear rate of 1,500 s-1 using a Wells-Brookfield cone-plate viscometer (DV3TLVCJ0, USA). All viscosity measurements were performed at 37°C.

### Biochemical analysis

#### Samples handling

Blood samples were centrifuged for 10 min at 3,000 x g to obtain the serum supernatant. The serum and tissue samples were stored at -80°C until the day of the experiment. The tissue samples were homogenized in PBS (phosphate-buffered saline, pH: 7.4) using a homogenizer (Fast prep-24, MP Biomedical, USA).

#### Analysis

Cholesterol, aspartate aminotransferase (AST), alanine aminotransferase (ALT), lipase and total protein (TP) levels in serum samples were measured with a semiautomatic biochemical analyzer (IDEXX Vettest, IDEXX Laboratories, Inc., USA). Detection ranges for the ALT, AST, lipase, cholesterol, and TP kits were 20–161 U/L, 39–111 U/L, 10–150 U/L, 20–92 mg/dl, and 5.3–6.9 g/dl, respectively.

### Analysis of oxidative stress parameters

#### Measurements of superoxide dismutase (SOD), malondialdehyde (MDA) and reduced glutathione (GSH) levels

SOD, MDA and GSH levels were measured with commercially available ELISA-based kits (for SOD, Elabscience Co., USA; for MDA, Mybiosource Co., USA; for GSH Elabscience Co., USA). Briefly, the standards and samples were pipetted into microplates coated with a monoclonal antibody and incubated. Biotin was added to all wells, and streptavidin-HRP was added to induce binding. After incubation, 4 washes were performed to remove unbound reagents. After adding chromogen solutions A and B, stop solution was added, and the resulting optical density was measured at 450 nm using a plate reader (Thermo Scientific, Varioskan™ LUX multimode microplate reader, USA). The detection range of the kits was between 0.16–10 ng/ml for SOD, 0–1000 ng/ml for MDA and 2–400 μmol/l for GSH.

#### Measurements of the total antioxidant status (TAS) and total oxidant status (TOS) levels

The TAS levels in the blood and tissue samples were measured using a commercial kit (Rel Assay Diagnostics, Turkey) with a spectrophotometric method (Thermo Scientific, Varioskan™ LUX multimode microplate reader, USA). Briefly, the antioxidants in the samples reduced the dark blue green 2,2′-azino-bis(3-ethylbenzothiazoline-6-sulfonic acid) diammonium salt (ABTS) radical to the colorless reduced form of ABTS. The change in absorbance at 660 nm was related to the total antioxidant level of the sample. Total antioxidant activities were reported as mmol Trolox equiv/L of the samples.

The TOS levels in the samples were measured using a commercial kit (Assay Rel Diagnostics, Turkey) and spectrophotometer (Thermo Scientific, Varioskan™ LUX multimode microplate reader, USA). Briefly, oxidants present in the samples oxidized the ferrous ion chelator complex to iron ions. While the oxidation reaction was prolonged by enhancer molecules abundant in the reaction medium, the ferric ion formed a color complex with chromogen in an acidic environment. The intensity of color formed was related to the total amount of oxidant molecules present in the samples. The results are reported as μm H_2_O_2_ equivalent/L.

### Measurement of inflammation parameters using multiplex ELISA

The inflammation parameters in serum samples were measured with a commercially obtained inflammation panel (Bio–Rad, Bioplex Rat cytokine plex assay, USA). The panel contains 30 parameters. The test was set up according to the manufacturer’s recommendations. Briefly, samples were mixed with antibody-bound magnetic beads in a 96-well plate and incubated overnight at 4°C with shaking. The cold and room temperature incubation steps were carried out on an orbital shaker at 500–600 x rpm. The microplate was washed twice with wash buffer. After a 1 h incubation at room temperature with the biotinylated detection antibody, streptavidin was added. The plate was washed, and PBS was added with a lower limit of 50 beads per sample. Reading was performed on a Bio-Plex 200 instrument (Bio–Rad, USA).

### Sodium dodecyl sulfate-polyacrylamide gel electrophoresis (SDS–PAGE) and western blot analysis

After thawing, the tissues (approximately 100 mg) were cut into pieces and homogenized in 500 μl of cold RIPA buffer (Santa Cruz Biotechnology, Dallas, Texas, USA) containing a protease inhibitor cocktail using a bead homogenizer for 10 min at a speed of 30/s. The homogenate was centrifuged at 700 × g for 15 min at 4°C to remove debris and nuclei, the supernatant was collected in a 1.5 ml tube, and groups were pooled. Samples were stored at -80°C until further experiments.

Supernatants of tissues were denatured and separated on a 12% sodium dodecyl sulfate-polyacrylamide gel. Proteins (20 μg) were transferred to a polyvinyl difluoride membrane (Bio–Rad, Hercules, CA, USA). The membrane was blocked with 5% nonfat dry milk (Bio–Rad, Hercules, CA, USA) in Tris (hydroxymethyl) aminomethane (Tris)-buffered saline containing 0.1% Tween 20 (TBST) for 1 h. The membrane was then immunoblotted with primary antibodies (IL-1β, TNF-α, Bax, Bcl-2, tubulin, and β-actin (Cell Signaling, Danvers, Massachusetts, USA)) overnight at 4°C. After three washes with the TBST solution, membranes were incubated with horseradish peroxidase (HRP)-conjugated secondary antibodies (goat anti-rabbit immunoglobulin G (IgG) HRP and goat anti-mouse IgG HRP (Cell Signaling, Danvers, Massachusetts, USA)) for 1 h at room temperature. Western blots were developed by a peroxidase reaction with ECL reagents (Elabscience, Houston, Texas, USA) and images were captured with a Fusion FX7 system (Vilber Lourmat, France). The band intensity was quantified using ImageJ software (National Institutes of Health, Bethesda, MD, USA).

### Statistical analysis

Group means ± standard deviations (SD) were calculated from all values. The Shapiro–Wilk test was used to test the normality of the data. Data with a normal distribution were analyzed with one-way ANOVA, and the data with a nonnormal distribution were analyzed with the Kruskal–Wallis test. Post hoc comparisons between the groups were performed with Bonferroni and Dunn tests using GraphPad Prism software (GraphPad Prism version 6 Software Program San Diego, CA). A value of P<0.05 was considered statistically significant.

## Results

### Number of culturable bacteria in blood

One hour after the LRW or *Staphylococcus aureus* injection, just before the electrical signal application, no bacterial growth was observed in the blood of the C and E groups, while there was dense bacterial growth was detected in the blood of the S and SE groups, which indicated bacteremia in these two groups. The 40-minute electrical signal application performed at 1 and 6 hours after the bacterial injection significantly (P<0.001) reduced bacteremia in the SE group compared to the S group (Fig 2). Slight bacteremia was still observed in the SE group.

### Tissue histology

**Fig 3:**
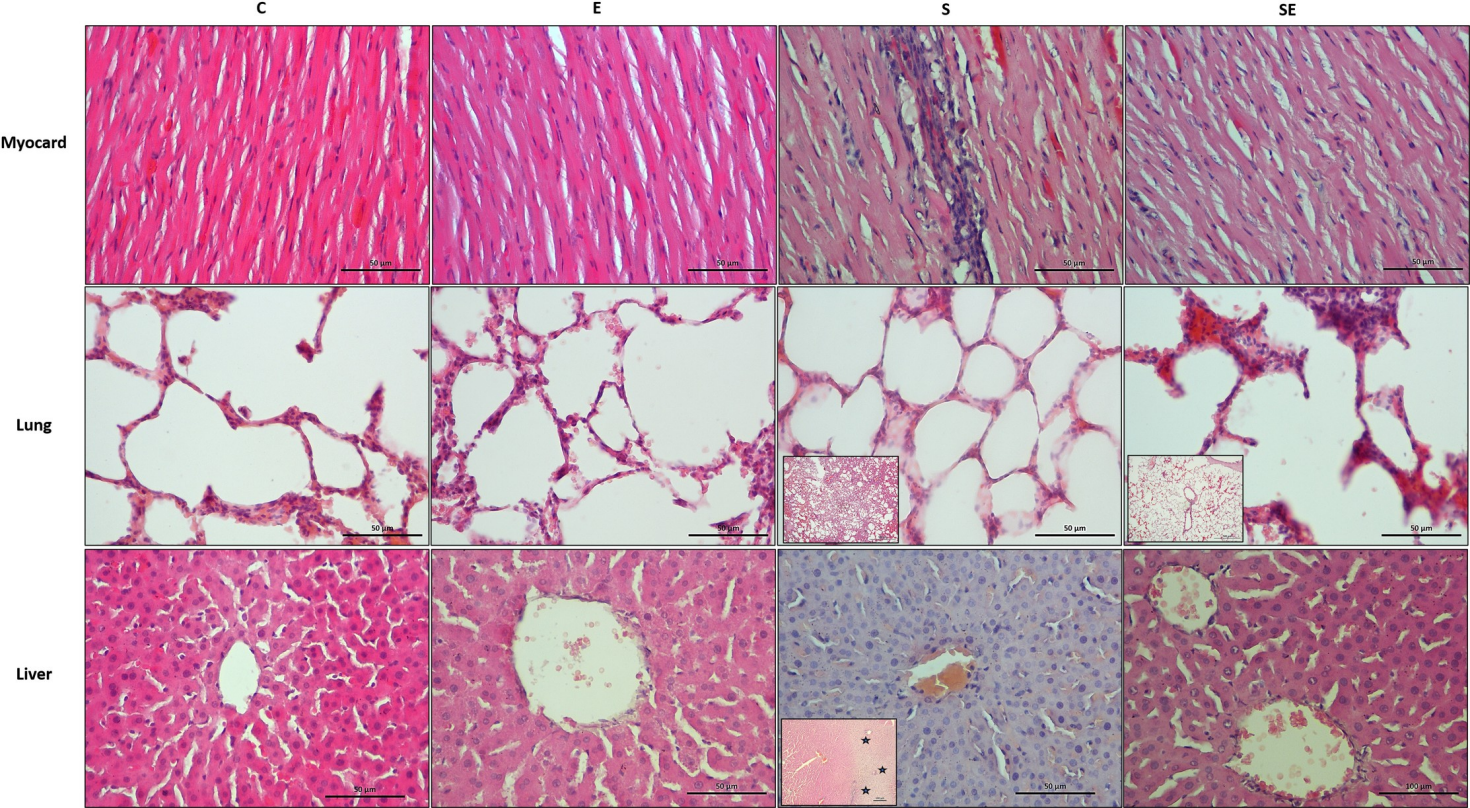
Histological results obtained after the Dr. Biolyse treatment. Images of hematoxylin & eosin staining in sections from the healthy control (C), electrified healthy (E), sepsis (S) and electrified sepsis (SE) groups at X400 magnification. Insets show the lung tissues of the S and SE groups at low magnification. Here, inflammation was greater in the S group than in the SE group (HE X10). Stars in insets in the pictures of the liver section from S group show the necrotic liver tissue (HE X10).

Histological results from the groups are shown in Fig 3. While inflammation was observed in the myocardium of the S group, no inflammation was observed in the myocardium of the SE group. Except for the S group, all groups had normal histological structures. Myocardial fiber bundles were loosely arranged, and some myocardial fibers were necrotic in the S group.

An examination of lung sections revealed that neither the C nor the E groups showed pulmonary histological alterations. Inflammatory cell infiltration was observed in the S group. Inflammation was also detected in the SE group, but it was much less than that of the S group. In addition, congestion was greater in the SE group than in all other groups.

Regarding liver tissues, no increase in inflammatory cell infiltration was observed in any group. Except for the S group, all groups had normal histological structures. However, sinusoids were slightly larger in all groups due to fixation. Areas of necrosis were observed in the tissues from the S group.

### Changes in blood parameters

**Fig 4:**
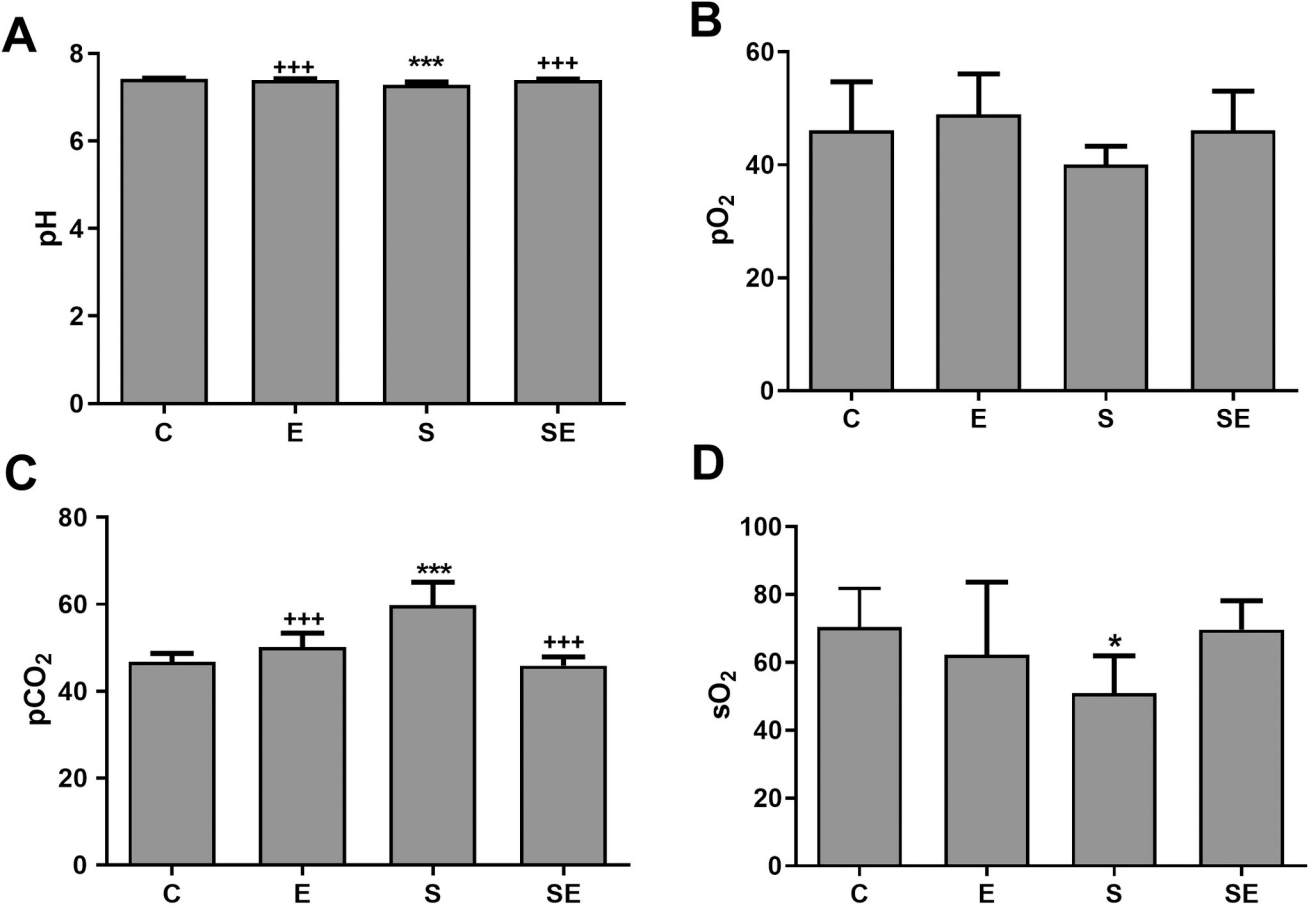
The blood parameters measured after the Dr. Biolyse treatment. The blood pH and gases of the healthy control (C) electrified healthy (E), sepsis (S) and electrified sepsis (SE) groups (n = 7). pH (A), pO_2_ (B), pCO_2_ (C) and sO_2_ (D) are presented as the means ± SD. *; *p*<0.05, ***; *p*<0.001 statistically significant difference compared to the C group. ^+++^; *p*<0.001 statistically significant difference compared to the S group.

**Fig 5:**
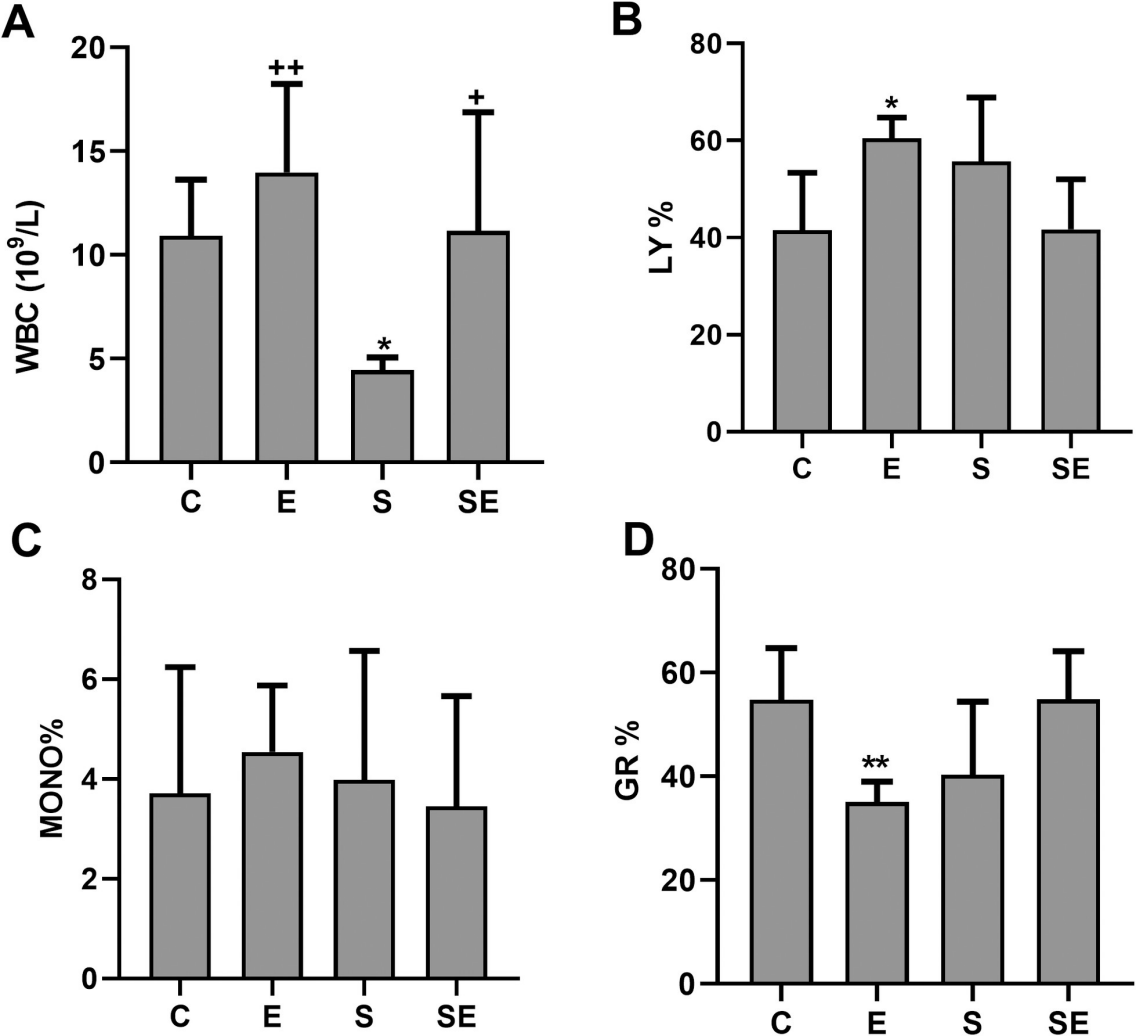
The white blood cell count. The blood leukocyte counts of the healthy control (C) electrified healthy (E), sepsis (S) and electrified sepsis (SE) groups (n = 7). WBC (A), white blood cells; LY% (B), lymphocyte %; MONO% (C), monocyte %; GR% (D), granulocyte %. Data are presented as the means ± SD. *; *p*<0.05, **; *p*<0.01 statistically significant difference compared to the C group. ^+^; *p*<0.05, ^++^; *p*<0.01 statistically significant difference compared to the S group.

**Fig 6:**
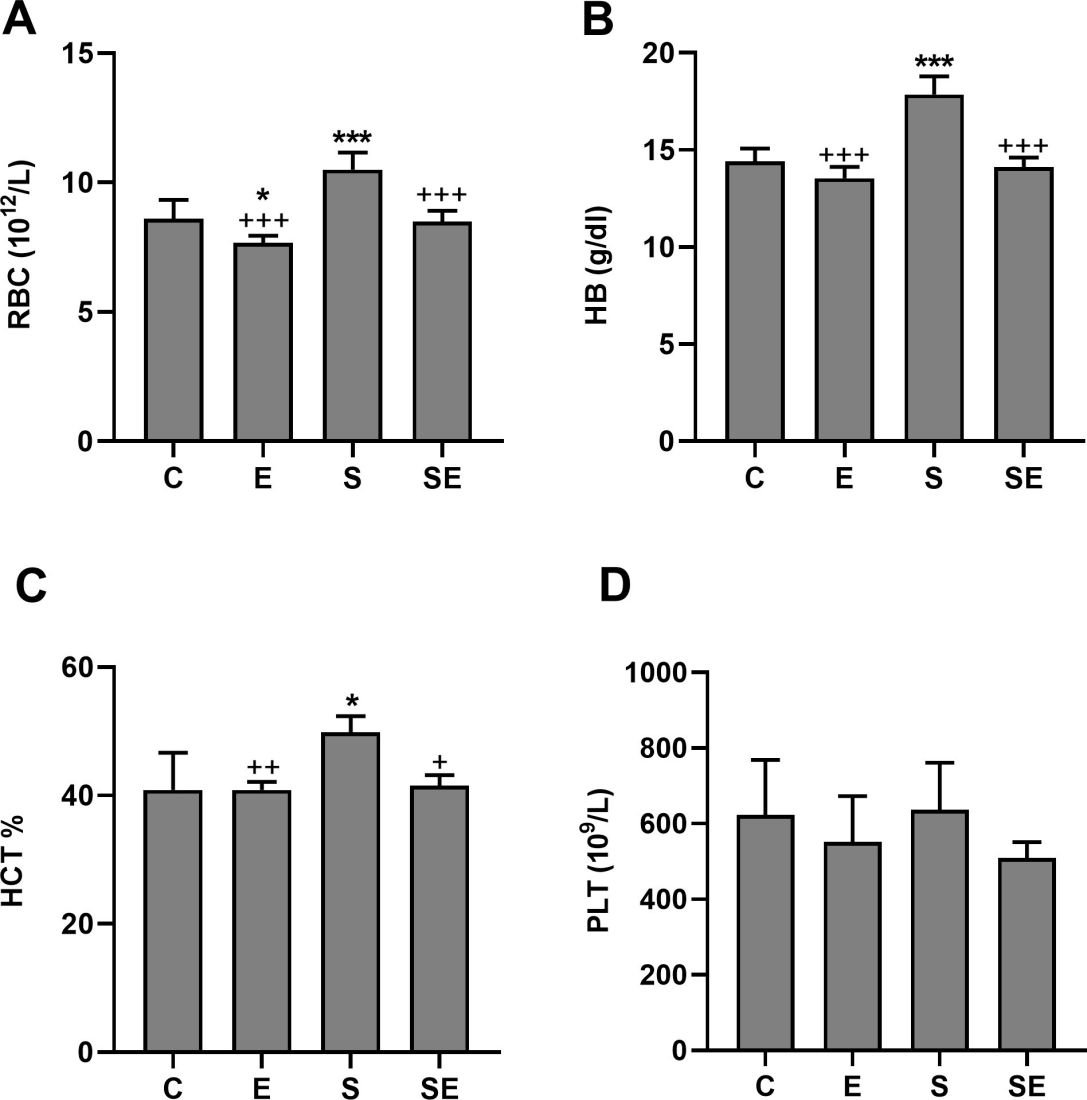
The blood hemogram parameters. The blood erythrocyte and thrombocyte counts and hemoglobin and hematocrit levels in the healthy control (C), electrified healthy (E), sepsis (S) and electrified sepsis (SE) groups (n = 7). RBC (A), red blood cell; HB (B), hemoglobin; HCT (C), hematocrit; and PLT (D), platelet. Data are presented as the mean ± SD. *; *p*<0.05, ***; *p*<0.001 statistical significance compared to the C group. ^+^; *p*<0.05, ^++^; *p*<0.01, ^+++^; *p*<0.001 statistical significance compared to the S group.

**Fig 7:**
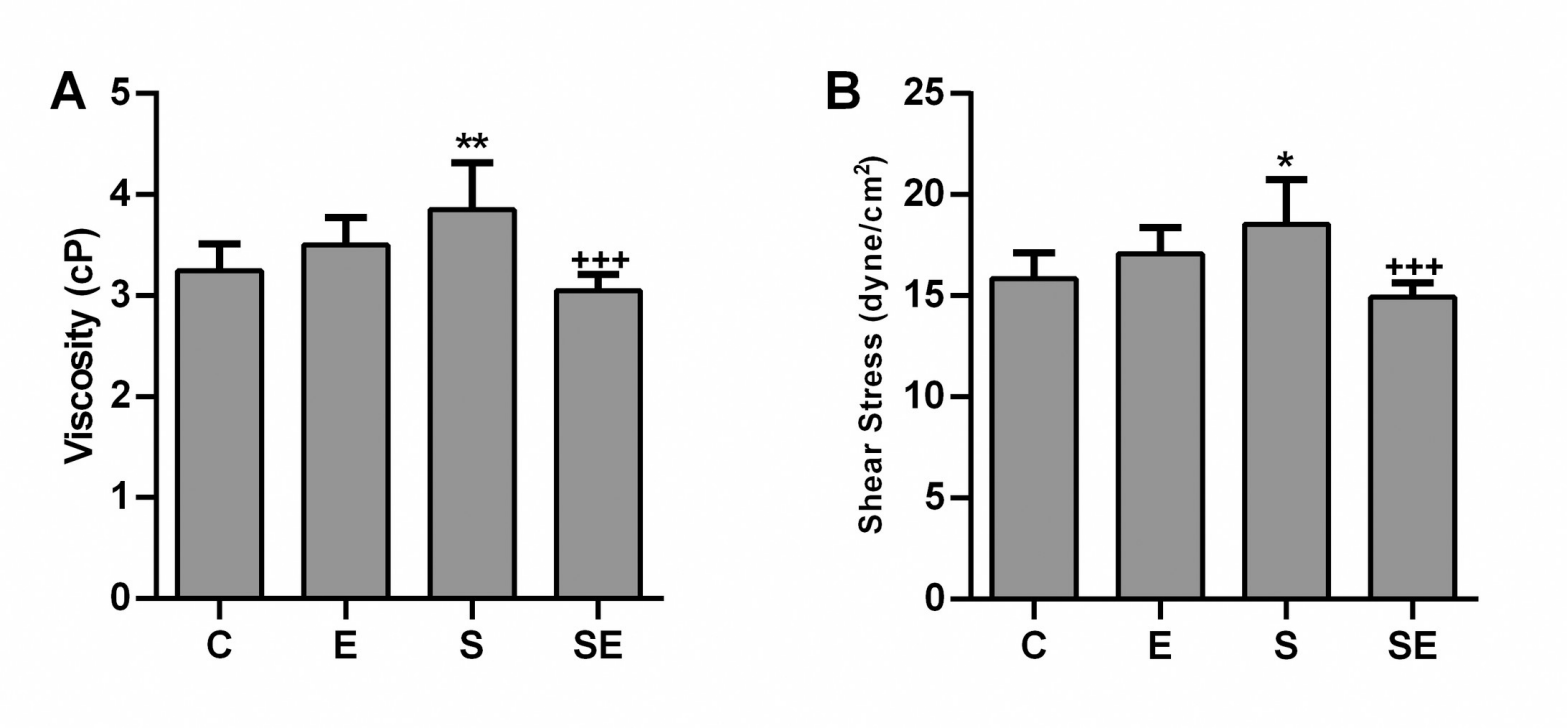
The blood rheological parameters. The blood viscosity and shear stress levels of the healthy control (C) electrified healthy (E), sepsis (S) and electrified sepsis (SE) groups (n = 7). Viscosity (A) and shear stress (B). Data are presented as the means ± SD. *; *p*<0.05, **; *p*<0.01 statistically significant difference compared to the C group. ^+++^; *p*<0.001 statistically significant difference compared to the S group.

Compared to the C group, the pH, oxygen saturation (sO_2_) and white blood cell (WBC) count were significantly lower (p<0.05—p<0.001), and the partial carbondioxide (pCO_2_), red blood cell (RBC) count, hemoglobin (HB) level, hematocrit percentage (HCT %), blood viscosity and shear stress levels were significantly higher (p<0.05—p<0.001) in the S group. However, none of the changes that occurred in the S group were observed in the SE group (Figs 4–7). Significant difference (p>0.05) among groups were not observed in platelet (PLT) counts, monocyte percentage or pO_2_. In addition, the lymphocyte percentage was significantly increased (p<0.05), and the granulocyte percentage and the RBC count were significantly decreased (p<0.01 and p<0.05, respectively) in the E group compared to the C group.

### Biochemical blood measurements

Compared to the C group, the serum levels of ALT and AST and lipase activity were significantly higher (p<0.01, p<0.01 and p<0.001, respectively), and the TP level was significantly lower (p<0.01) in the S group. However, none of the changes that occurred in the S group were observed in the SE group (Table 1).

**Table 1:** The serum ALT, AST, cholesterol and TP levels and lipase activity and the blood and tissue TAS, TOS, MDA and GSH levels and SOD activity.

**Serum**	**Groups**			
	**C**	**E**	**S**	**SE**
**AST (U/L)**	53.14±2.73	53.17±4.84^**++**^	79.14±28.13**	58±9.03^**+**^
**ALT (U/L)**	93.29±5.4	92.42±3.8^**+++**^	186.7±94.65**	98±15.19^**++**^
**TP (g/dl)**	5.8±0.5	5.817±0.16^**+++**^	4.786±0.49**	5.314±0.65
**CHOLESTEROL (mg/dl)**	51±3.05	52.25±5.11	35.71±20.66	44.86±20.36
**LIPASE (U/L)**	62.14±37.72	58.25±12.56^**+++**^	1419±1123***	60±37^**+++**^
**TAS (mmol/L)**	1.2±0.14	1.27±0.16^**+++**^	0.88±0.09***	1.25±0.13^**+++**^
**TOS (μmol/L)**	4.41±0.62	3.75±0.34^**+++**^	8.51±1.99***	4.5±0.71^**+++**^
**SOD (ng/ml)**	9.32±1.11	9.9±1.53^**+++**^	4.94±0.73***	9.13±1.29^**+++**^
**MDA (ng/ml)**	142±37.81	119.9±28.06^**+++**^	784.4±143.2***	200±53.01^**+++**^
**GSH (μmol/L)**	280.2±52.11	289.2±52.4^**+++**^	72.54±18.67***	281.6±49.57^**+++**^
**Lung**	**Groups**			
	**C**	**E**	**S**	**SE**
**TAS (mmol/L)**	1.21±0.09	1.26±0.09^**+++**^	0.84±0.11***	1.25±0.06^**+++**^
**TOS (μmol/L)**	4.39±0.61	3.71±0.33^**+++**^	8.53±1.92***	4.55±0.61^**+++**^
**SOD (ng/ml)**	10.36±1.59	11.68±1.03^**+++**^	5.96±0.68***	9.93±0.92^**+++**^
**MDA (ng/ml)**	224.8±77.97	209.9±49.10^**+++**^	1146±350***	372.6±67.84^**+++**^
**GSH (μmol/L)**	291.9±45.29	304.6±59.91^**+++**^	98.67±16.71***	282.8±16.62^**+++**^
**Liver**	**Groups**			
	**C**	**E**	**S**	**SE**
**TAS (mmol/L)**	1.27±0.09	1.3±0.09^**+++**^	0.87±0.12***	1.29±0.06^**+++**^
**TOS (μmol/L)**	4.54±0.63	3.83±0.34^**+++**^	8.82±1.99***	4.7±0.63^**+++**^
**SOD (ng/ml)**	10.66±0.85	12.28±1.34* ^**+++**^	5.98±0.69***	9.22±1.05^**+++**^
**MDA (ng/ml)**	229.4±56.67	220.1±47.63^**+++**^	1215±115.1***	238.7±33.49^**+++**^
**GSH (μmol/L)**	310.3±69.59	341.6±69.66^**+++**^	132.9±41.61***	308.9±24.62^**+++**^

Data are presented as the means ± SD.

*; p<0.05

***; p<0.001 statistically significant difference compared to the C group.

+++; p<0.001 statistically significant difference compared to the S group (n = 7).

### Oxidative stress analysis

Compared to the C group, the blood and tissue levels of TOS and MDA were significantly higher (p<0.001), and TAS and GSH levels and SOD activity were significantly lower (p<0.001) in the S group. However, none of the changes that occurred in the S group were observed in the SE group (Table 1).

### Analysis of cytokine levels in the blood

The serum inflammation markers are shown in Table 2. The levels of all examined interleukins (ILs) and macrophage inflammatory proteins (MIPs), except for IL-5, IL-13 and MIP-3α, were significantly increased (p<0.05 –p<0.001) in the S group compared to the C group. In addition, granulocyte colony-stimulating factor (G-CSF) and macrophage colony-stimulating factor (M-CSF) levels were significantly lower (p<0.05 and p<0.001, respectively), and granulocyte-macrophage colony-stimulating factor (GM-CSF), growth-regulated oncogenes (GRO)/keratinocyte chemoattractant (KC), interferon gamma (IFNγ), monocyte chemoattractant protein 1 (MCP1) and chemokine ligand (CCL5 or RANTES) levels were significantly higher (p<0.001) in the S group than in the C group. However, all these parameters that changed in the S group, except for IL-1α, were unchanged in the SE group. The level was still higher (p<0.05) in the SE group than in the C group.

**Table 2:** The serum inflammation markers.

Inflammation Markers	Groups			
	C	E	S	SE
**G-CSF (pg/ml)**	2,51±0,57	4,06±1.17** ^**+++**^	1,22±0.59*	2,79±0.62^**++**^
**M-CSF (pg/ml)**	19,97±4.68	18,7±5.02^**+++**^	6,62±1.96***	17,39±5.8^**++**^
**GM-CSF (pg/ml)**	7,49±2.97	8,07±2.79^**+++**^	16,75±3.1***	9,43±2.5^**+++**^
**GRO/KC (pg/ml)**	2,47±1.17	2,8±1.17^**+++**^	5,08±1.04***	3,17±0.84^**+**^
**IFNγ (pg/ml)**	24,23±9.61	26,11±9.05^**+++**^	50,72±7.26***	29,07±5.84^**+++**^
**MCP1 (pg/ml)**	28,49±4.52	25,09±6.83^**+++**^	83,13±14.78***	29,96±4.57^**+++**^
**IL-1α (pg/ml)**	19,15±4.52	18,88±6.76^**+++**^	48,11±5.47***	28,75±7.54* ^**+++**^
**IL-1β (pg/ml)**	15,96±2.84	14,78±5.51^**+++**^	31,82±5.34***	15,54±5.07^**+++**^
**IL-2 (pg/ml)**	25,7±7.17	25,55±7.13^**+++**^	56,83±7.73***	30,44±6.85^**+++**^
**IL-4 (pg/ml)**	5,8±0.65	5,3±1.42^**+++**^	12,07±2.52***	6,04±1.93^**+++**^
**IL-5 (pg/ml)**	20,3±4.35	21,86±6.24	20,04±8.26	18,35±7.15
**IL-6 (pg/ml)**	24,87±3.7	20,53±5.38^**+++**^	63,73±6.57***	23,58±6.59^**+++**^
**IL-7 (pg/ml)**	6,97±1.41	6,88±0.94^**+++**^	13,1±1.63***	8,5±1.81^**+++**^
**IL-10 (pg/ml)**	13,4±2.45	12,81±3.3^**+++**^	30,7±3.95***	15,25±3.27^**+++**^
**IL-12 p40 (pg/ml)**	10,62±1.99	9,7±1.59^**++**^	16,65±6.45*	13,85±4.36
**IL-12 p70 (pg/ml)**	15,71±2.64	14,47±2.7^**+++**^	30,44±4.78***	17,56±3.31^**+++**^
**IL-13 (pg/ml)**	13,07±4.36	14,62±6.24	12,81±8.26	11,11±7.15
**IL-17A (pg/ml)**	5,34±1.64	4,75±1.33^**+++**^	12,74±2***	7,75±3.01^**+++**^
**IL18 (pg/ml)**	34,91±6.38	33,08±7.12^**+++**^	70,83±7.49***	38,74±8.21^**+++**^
**MIP-1α (pg/ml)**	23,59±5.57	23,27±8.33^**+++**^	63,86±6.22***	32,58±6.12^**+++**^
**MIP-2 (pg/ml)**	1,18±0.45	1,13±0.59^**+++**^	2,52±0.41***	1,32±0.47^**+++**^
**MIP-3α (pg/ml)**	3,08±1.02	3,45±1.47	3,02±1.95	2,62±1.69
**RANTES (pg/ml)**	10,8±1.45	10,3±3.21^**+++**^	24,8±3.99***	13,87±2.68^**+++**^

Data are presented as the means ± SD.

*; p<0.05

***; p<0.001 statistically significant difference compared to the C group.

+++; p<0.001 statistically significant difference compared to the S group (n = 7).

### Western blotting

**Fig 8:**
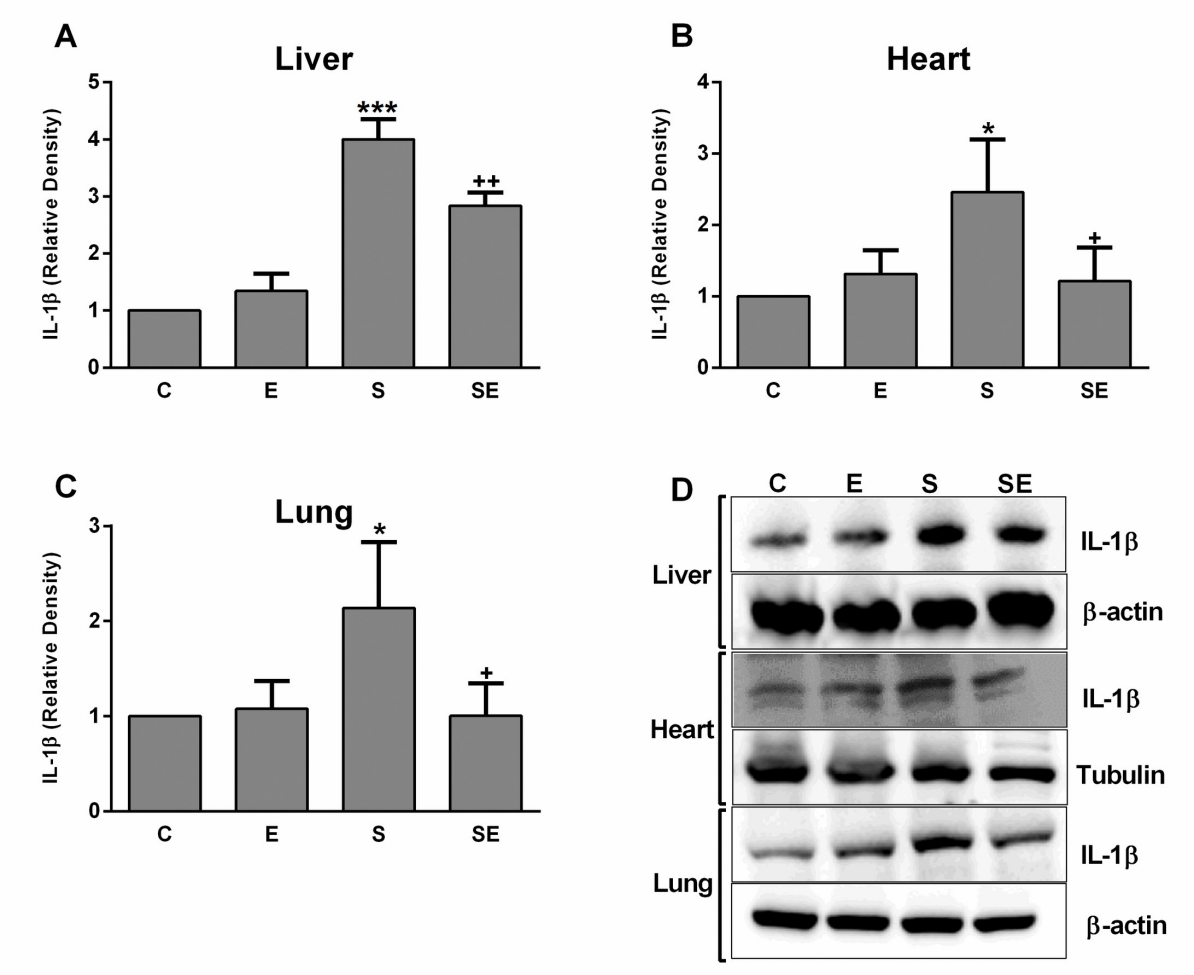
Relative protein expression of the proinflammatory cytokine IL-1β. A) Images of liver, B) heart, and C) lung tissues from healthy control (C), electrified healthy (E), sepsis (S) and electrified sepsis (SE) groups. D) Representative images of IL-1β expression in the studied tissues. Values are presented as the means ± SD. Comparisons were performed between the C group and the S group (*; *p*<0.05 and ***; *p*<0.001) and between the S group and the SE group. (^+^; *p*<0.05 and ^++^; *p* <0.01).

**Fig 9:**
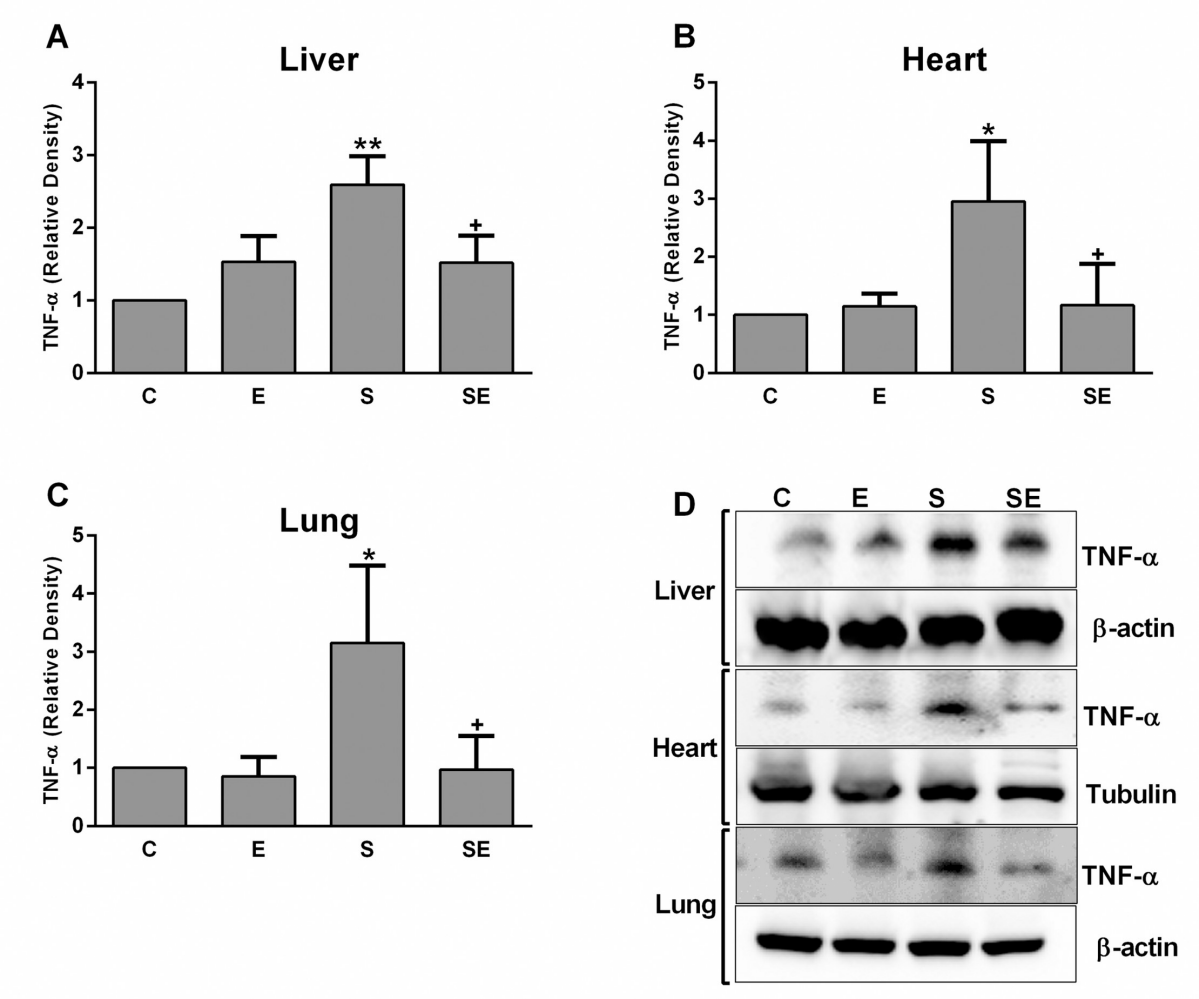
The relative protein expression of the proinflammatory cytokine TNF-α. A) Images of the liver, B) heart, and C) lung tissues from the healthy control (C), electrified healthy (E), sepsis (S) and electrified sepsis (SE) groups. D) Representative pictures of TNF-α expression in the studied tissues are shown. Values are presented as the means ± SD. Comparisons were performed between the C group and the S group (*; *p*<0.05 and **; *p*<0.01) and between the S group and the SE group. (^+^; *p*<0.05).

Changes in the levels of the inflammatory cytokines IL-1β and TNF-α in the liver, heart and lung tissues of rats are shown in Figs 8 and 9. In all examined tissues, the expression of these cytokines was upregulated (p <0.05 –p<0.001) in the S group compared to the C group. The electrical signal treatment significantly decreased (p<0.05 –p<0.01) the levels of both IL-1β and TNF-α in the SE group compared to the S group. In addition, electrical signal treatment of healthy rats did not induce any change in the expression of these inflammatory cytokines in the liver, heart, or lung tissues.

**Fig 10:**
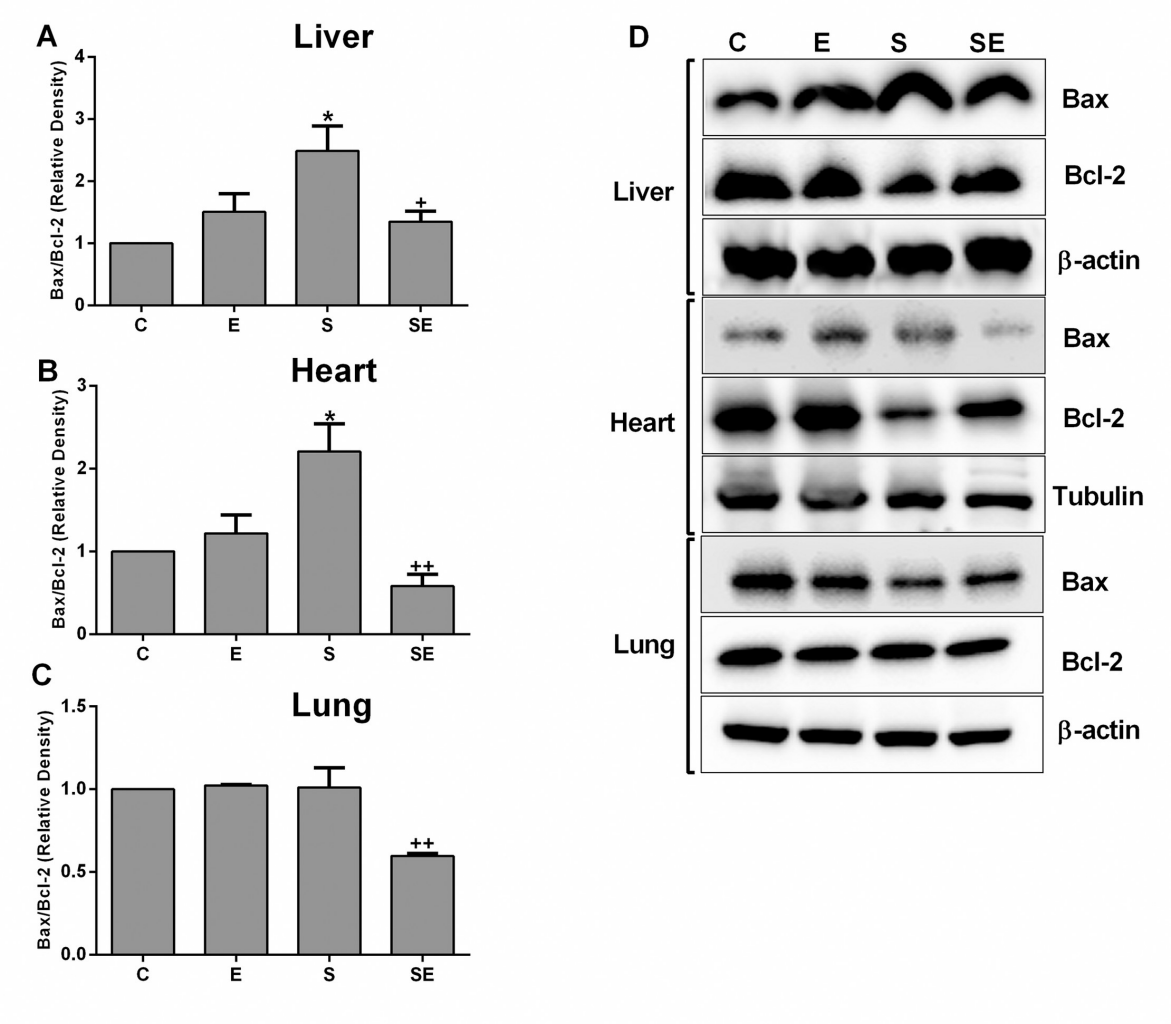
The relative ratio of expression of the proapoptotic protein Bax to the antiapoptotic protein Bcl-2. A) Data from the liver, B) heart, and C) lung tissues from the healthy control (C), electrified healthy (E), sepsis (S) and electrified sepsis (SE) groups. D) Representative pictures of Bax and Bcl-2 expression in the studied tissues. Values are presented as the means ± SD. Comparisons were performed between the C group and the S group (*; *p*<0.05) and between the S group and the SE group. (^+^; *p*<0.05 and ^++^; *p*<0.01).

Due to the relationship between the increased levels of proinflammatory cytokines and apoptosis during the process of sepsis, the ratio of Bax (a proapoptotic member of the Bcl-2 family) to Bcl-2 (an antiapoptotic member of the Bcl-2 family) expression was analyzed as a marker for the rate of apoptosis (Fig 10). In the liver and heart tissues, the Bax/Bcl-2 ratio was significantly higher (p<0.05) in the S group than in the C group, indicating an increase in apoptosis. However, the Bax/Bcl-2 ratios in the lung tissues from the S group were not changed compared to the C group. The electrical signal treatment significantly decreased (p<0.05 –p<0.01) the apoptosis rate in the liver, heart and lung tissue of the SE group compared to the S group. In addition, electrical signal treatment of the healthy rats did not produce any alteration in apoptosis in the examined tissues compared to the C group.

## Discussion

The antibacterial effect of a low direct electrical signal on experimentally induced septic rats *in vivo* was first described in our study. We proposed that the antibacterial effect of electrical stimulation might be due primarily to disrupting the integrity of the bacterial membrane through the electrolysis of molecules on the bacterial cell surface [15, 16] or secondarily to the formation of HOCl due to the electrolysis of body fluids by a low electrical signal. When in contact with microbes, HOCl selectively binds the unsaturated lipid layer of microorganisms and then disrupts their cell wall and thus the cellular integrity of microbes or viruses, causing them to be destroyed [20]. In addition, HOCl is also produced by activated neutrophils in the immune response to invading pathogens and immediately reacts with proteins mostly belonging to the host [21, 22]. The exposure of human monocyte-derived macrophages to HOCl increases the intracellular Ca^2+^ concentration, which might be the pathway by which leukocytes form extracellular traps [23]. Similarly, the release of extracellular traps occurs when neutrophils are exposed to the Ca^2+^ ionophore ionomycin [23].

Histologically, the preservation of tissue integrity and the absence of necrosis showed that low direct electrical signal application did not cause any damage to the rat tissues or organs. Although inflammation was observed in the lung tissues from both the S and SE groups, it was much less common in the SE group than in the S group. Thus, the rats were successfully infected experimentally, but the application of the low direct electrical signal significantly reduced the spread of infection in lung tissue.

In our study, the pH, sO_2_ and WBC count were lower, and the pCO_2_, RBC count, HB, HCT %, blood viscosity and shear stress levels were higher in the S group than in the C group. Similar to our result, blood pH decreases in individuals with severe sepsis [24, 25]. The changes in the pH and blood gas levels in the S group might be due to the association of sepsis with multiple organ failure that might lead to respiratory and circulatory system failure and acute kidney injury [26, 27].

Blood viscosity is a measure of the thickness of blood and is defined as the ratio between the shear stress and shear rate. It may increase in individuals with acute inflammatory diseases due to the increase in acute phase proteins [28]. HCT % may also alter the blood viscosity. In our study, the increased blood viscosity observed in the septic rats compared to the controls was probably due to the increased RBC count, HB level, and HCT %. In patients with sepsis, respiratory (hypoventilation) and circulatory (hypoperfusion) failures may lead to tissue hypoxia [29]. Therefore, hypoxia-induced erythropoietin production might explain the increased RBC count, HB value, and HCT % in the septic rats compared to the controls. The WBC count decreased significantly in the S group compared to the C group. A decreased WBC count may be associated with immune paralysis. It represents an important feature of severe sepsis and causes an increased mortality rate [30]. However, none of the changes that occurred in the S group were observed in the SE group. In addition, the low electrical signal increased the lymphocyte percentage and decreased the granulocyte percentage and the RBC count in the E group compared to the C group. Our experiment is the first to document this effect of a low electrical signal on lymphocyte and monocyte percentages.

The serum levels of ALT and AST and lipase activity were higher, and the TP level was lower in the S group than in the C group. Increased lipase activity is an indicator of pancreatitis or septic shock [31], and increased levels of ALT and AST and decreased levels of TP are markers of liver damage [32]. The application of the low electrical signal prevented the occurrence of all these changes in the SE group.

Previous studies have revealed that lung and kidney injuries occur [33, 34] due to excessive production of reactive oxygen species (ROS), such as MDA, in tissues, neutrophil accumulation and an increase in proinflammatory cytokine production [35, 36] during sepsis. ROS levels are maintained in balance because they are neutralized by the body’s antioxidant defense systems, such as GSH and SOD [37]. If this balance is disrupted in favor of ROS, destructive reactions occur in molecules such as proteins, lipids, and nucleic acids. This condition, called "oxidative stress", ultimately leads to tissue damage [38]. The blood and tissue levels of TOS and MDA were higher, and the TAS and GSH levels and SOD activity were lower in the S group than in the C group, indicating a disrupted oxidant/antioxidant balance in favor of oxidants and thus oxidative stress, as observed in other inflammatory diseases, such as osteoarthritis and pulmonary fibrosis [39, 40]. However, the application of the low electrical signal prevented the formation of oxidative stress in the SE group, indicating an antioxidant effect.

The levels of many ILs increase in individuals with sepsis [41–43]. Parallel to previous studies, the levels of all examined ILs, except IL-5 and IL-13, increased in the S group compared to the C group in our study. A low level of IL-5 is associated with lung death and tissue damage, and thus, IL-5 treatment may reduce the mortality associated with sepsis [44]. In a previous study, IL-13 protected against sepsis-induced lethality by suppressing inflammatory responses [45]. As shown in our study, the levels of all examined MIPs, except MIP-3α, increased in the S group compared to the C group. In the literature, limited information is available about the MIP-3α level, and the roles of other MIP families in sepsis are controversial [46, 47]. The use of G-CSF, the levels of which were decreased in the S group, is recommended in patients with sepsis to increase myeloid cell functions [48]. Interestingly, while the GM-CSF level was expected to decrease in the S group [49], it was higher than that in the controls in our study. This change may be related to the early or late stage of sepsis. The GRO/KC, MCP1 and RANTES levels were higher in the S group than in the C group in our study. To our knowledge, no study has assessed the GRO level in individuals with sepsis, but there are many studies on patients with various cancers [50, 51]. GRO levels have been reported to increase in individuals with different types of cancer. MCP1 is a potent chemoattractant and a regulatory mediator involved in various inflammatory diseases [52]. RANTES also exerts a similar effect [53]. However, the application of electrical signals prevented the formation of inflammatory markers, except IL-1α, in the E group, indicating an anti-inflammatory effect. IL-1α is produced by activated macrophages and neutrophils, epithelial cells, and endothelial cells. It plays important roles in regulating immune responses by binding the interleukin-1 receptor [54]. The higher IL-1 level in the SE group than in the C group might be due to the presence of slight bacteremia in the SE group. In our study, a low electrical signal was applied in two sessions of forty minutes. Most likely, bacteremia would be eliminated by increasing the number of sessions. This finding also explains the presence of minor inflammation in the lungs of the SE group.

The relationship between sepsis and increased levels of proinflammatory cytokines such as TNF-α and IL-1β is known as a mechanism for eliminating invading pathogens [55, 56]. On the other hand, these immune system regulators have a role in sepsis-induced pathophysiology by promoting excessive tissue-damaging inflammation [57]. In the present study, upregulation of both TNF-α and IL-1β, the most extensively studied cytokines in sepsis pathophysiology, was noted in the liver, heart and lung tissues of rats in the S group, consistent with previous reports from other researchers [58–60]. The production of proinflammatory cytokines is strongly related to a high level of ROS generation in subjects with sepsis [61, 62]. However, the low electrical signal decreased the levels of these cytokines and oxidative stress parameters to levels similar to the control group by improving sepsis-induced tissue damage. In a previous electroacupuncture study, the sepsis-induced increase in proinflammatory cytokine levels in the lung tissue was also decreased by electrical stimulation [63]. In addition, researchers also found that electrical vagus nerve stimulation during sepsis reduced the level of TNF-α by activating an anti-inflammatory mechanism [64]. In addition, our newly developed low direct electrical signal application method may also enhance an anti-inflammatory mechanism as compensation for sepsis-induced tissue damage.

During sepsis, the rate of cellular death increases due to the activation of the mitochondrial apoptosis pathway [65]. In the current study, we also observed an increase in the apoptotic rate in liver and heart tissues, consistent with recent studies [59, 66]. The induction of the pathophysiological process of sepsis may result from both an apoptosis-induced decrease in the number of immune cells and an immunosuppressive effect of apoptotic cells [65]. According to previous studies, inhibition of apoptosis might be an effective strategy to protect against sepsis-induced tissue damage [67]. Furthermore, we observed that the low electrical signal also decreased the rate of apoptosis to increase the chance of cell survival after sepsis. Xie et al. also reported a positive effect of electrical stimulation on the pulmonary expression of caspase-3 and Bax as a compensatory mechanism against apoptosis [63].

In summary, a low direct electrical signal application, without causing any damage to tissues or organs of rats has

- an antibacterial effect either disrupting the integrity of the bacterial membrane by the electrolysis of molecules or the formation of HOCl due to electrolysis of body fluids,
- an antioxidant effect by balancing disrupted oxidant/antioxidant status and decreasing oxidative stress,
- an anti-inflammatory effect by reducing the bacteremia, and thus inhibiting the formation of inflammatory markers,
- an antiapoptotic effect by adjusting the balance between proapoptotic (bax) and antiapoptotic (bcl-2) proteins in favor of antiapoptotic (bcl-2) proteins to reduce sepsis-dependent cell death.

Due to its anti-bacterial, antioxidant, anti-inflammatory and anti-apoptotic effects, Dr. Biolyse can offer additional benefits to the current therapies against to infectious diseases.

## Supporting information

S1 Raw data Raw data Table 1. (XLSX)

S2 Raw data Raw data Table 2. (XLSX)

S3 Raw data Raw data-Figs 4–7. (XLSX)

S1 Raw images (PDF)
