# Supplementary material for: A low direct electrical signal attenuates oxidative stress and inflammation in septic rats
Source: PLoS One. 2021 Sep 9;16(9):e0257177. doi: 10.1371/journal.pone.0257177 (PMC8428794; doi:10.1371/journal.pone.0257177)
Supplement: S1 Raw images — (PDF) [file pone.0257177.s004.pdf]

Fig 8. The relative protein expression of proinflammatory cytokine IL-1β.

(Healthy control (C), electrified healthy (E), sepsis (S) and electrified sepsis (SE) groups).

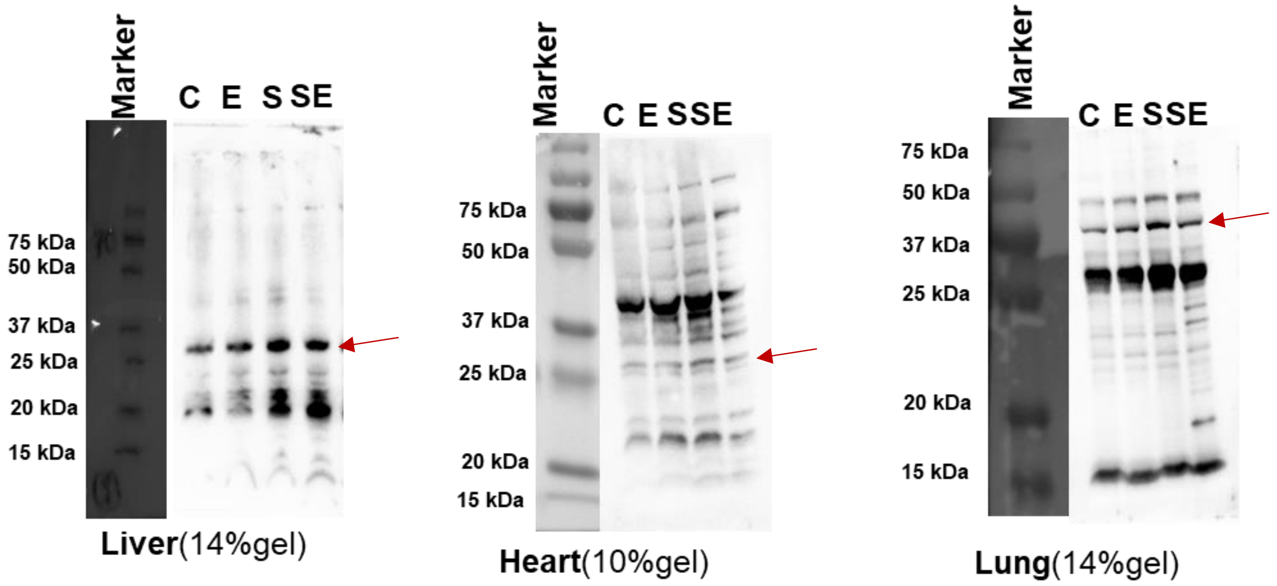

Fig 9. The relative protein expression of proinflammatory cytokine TNF-α.

(Healthy control (C), electrified healthy (E), sepsis (S) and electrified sepsis (SE) groups)

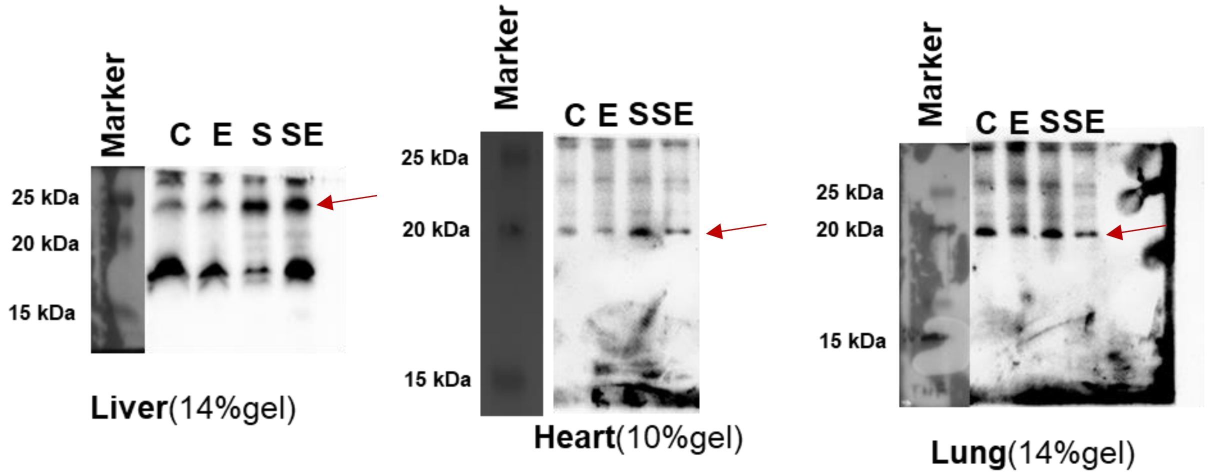

Fig 10. The relative protein expression ratio of pro-apoptotic protein Bax to the anti-apoptotic protein Bcl-2.

(Healthy control (C), electrified healthy (E), sepsis (S) and electrified sepsis (SE) groups).

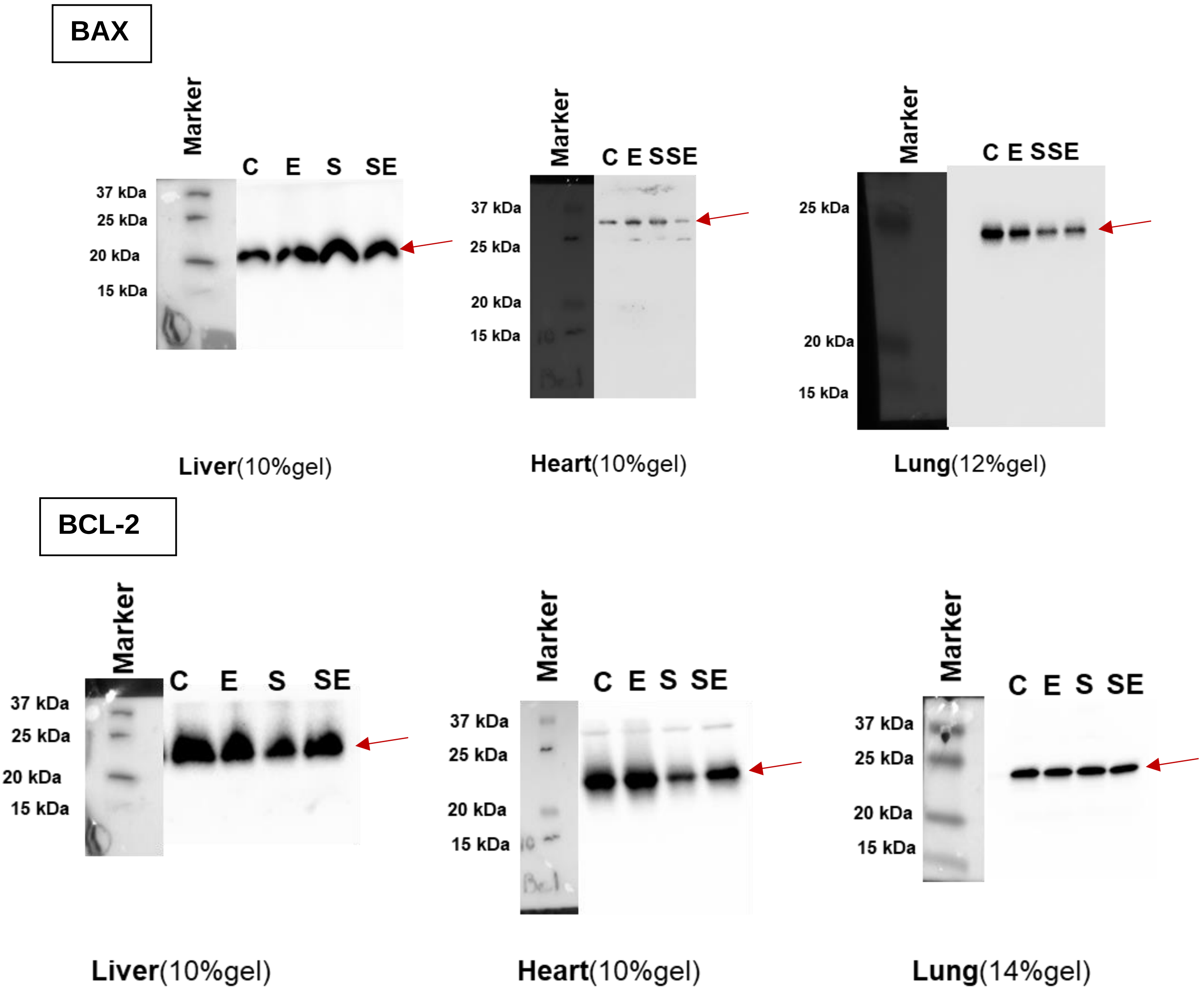

We performed imaging membranes with luminol substrate and captured by Fusion FX7 system (Vilber Lourmat, France).
